# Bone Regeneration by Nanohydroxyapatite/Chitosan/Poly(lactide-co-glycolide) Scaffolds Seeded with Human Umbilical Cord Mesenchymal Stem Cells in the Calvarial Defects of the Nude Mice

**DOI:** 10.1155/2015/261938

**Published:** 2015-10-13

**Authors:** Fei Wang, Xiao-Xia Su, Yu-Cheng Guo, Ang Li, Yin-Cheng Zhang, Hong Zhou, Hu Qiao, Li-Min Guan, Min Zou, Xin-Qin Si

**Affiliations:** ^1^Department of Orthodontics, Stomatological Hospital, College of Medicine, Xi'an Jiaotong University, Xi'an 710004, China; ^2^Research Center for Stomatology, Stomatological Hospital, College of Medicine, Xi'an Jiaotong University, Xi'an 710004, China; ^3^Department of Oral and Maxillofacial Surgery, Stomatological Hospital, College of Medicine, Xi'an Jiaotong University, Xi'an 710004, China

## Abstract

In the preliminary study, we have found an excellent osteogenic property of nanohydroxyapatite/chitosan/poly(lactide-co-glycolide) (nHA/CS/PLGA) scaffolds seeded with human umbilical cord mesenchymal stem cells (hUCMSCs) *in vitro* and subcutaneously in the nude mice. The aim of this study was to further evaluate the osteogenic capacity of nHA/CS/PLGA scaffolds seeded with hUCMSCs in the calvarial defects of the nude mice. Totally 108 nude mice were included and divided into 6 groups: PLGA scaffolds + hUCMSCs; nHA/PLGA scaffolds + hUCMSCs; CS/PLGA scaffolds + hUCMSCs; nHA/CS/PLGA scaffolds + hUCMSCs; nHA/CS/PLGA scaffolds without seeding; the control group (no scaffolds) (*n* = 18). The scaffolds were implanted into the calvarial defects of nude mice. The amount of new bones was evaluated by fluorescence labeling, H&E staining, and Van Gieson staining at 4 and 8 weeks, respectively. The results demonstrated that the amount of new bones was significantly increased in the group of nHA/CS/PLGA scaffolds seeded with hUCMSCs (*p* < 0.01). On the basis of previous studies *in vitro* and in subcutaneous implantation of the nude mice, the results revealed that the nHA and CS also enhanced the bone regeneration by nHA/CS/PLGA scaffolds seeded with hUCMSCs in the calvarial defects of the nude mice at early stage.

## 1. Introduction

Scaffold materials for bone tissue engineering are usually divided into inorganic materials, natural biomaterials, and synthetic polymer materials [1–3]. nHA [4], CS [5], and PLGA [6] are the representatives for the three kinds of materials, respectively, in bone tissue engineering. Therefore, in the preliminary study, we designed nHA/CS/PLGA scaffolds and preliminarily proved that the nHA/CS/PLGA scaffolds showed the higher compression and tensile modulus compared with the nHA/PLGA, CS/PLGA, and PLGA scaffolds (*p* < 0.05) [7].

In recent years, it has been reported that hUCMSCs have similar characteristics with bone marrow-derived mesenchymal stem cells (BMSCs). It might be used as a selection of seed cells for bone tissue engineering [8, 9].

In our preliminary study, when hUCMSCs were seeded onto nHA/CS/PLGA scaffolds the results showed higher osteoinduction activity compared with hUCMSCs seeded onto nHA/PLGA, CS/PLGA, or PLGA scaffolds* in vitro* and subcutaneously in the nude mice [7]. It was implied that PLGA combined with CS and nHA might result in an acceleration of osteogenic differentiation for hUCMSCs.

Nevertheless, the capacity of repairing bone defects is a key factor to assess the osteogenic ability in bone tissue engineering. Calvarial defect models of nude mice are used commonly in bone tissue engineering [10–13]. The present study was designed to make further evaluation on the effects of nHA/CS/PLGA scaffolds seeded with hUCMSCs on repairing the calvarial defects of nude mice.

## 2. Materials and Methods

### 2.1. Preparation of nHA/CS/PLGA Scaffolds

nHA/CS/PLGA scaffolds were prepared as described in the preliminary study [7]. Briefly, nHA (particle size (XRD) ≤ 40 nm, Nanjing Emperor Nano Material Co., Ltd.), CS (degree of deacetylation: 85%~95%, Zhejiang Golden-Shell Biochemical Co., Ltd.), and PLGA (PLA/PGA: 80 : 20, glass transition temperature: 45~55°C, viscosity of IV (dL/g): 0.15~3.0, Jinan Daigang Biomaterial Co., Ltd.) were prepared in the weight ratio of 10 : 10 : 80. PLGA was dissolved in chloroform to the concentration of 10%. nHA and CS were added and mixed fully after PLGA was completely dissolved. And then, sodium chloride was added into the solutions in the volume ratio of 1 : 10. The particle sizes of sodium chloride were between 150 and 250 *μ*m. The solutions were mixed uniformly. After ultrasonic degassing, the solutions were poured into the molds and stood for 24–48 h. And then, the solutions were hardened and the scaffolds formed. The scaffolds were demolded and dried for use. At the same time, nHA/PLGA (weight ratio: 20 : 80), CS/PLGA (weight ratio: 20 : 80), and PLGA scaffolds were prepared.

### 2.2. nHA/CS/PLGA Scaffolds Seeded with hUCMSCs* In Vitro*

hUCMSCs were obtained as described in the preliminary study [7]. hUCMSCs at P3 were seeded onto PLGA, nHA/PLGA, CS/PLGA, and nHA/CS/PLGA scaffolds, respectively, and maintained in an osteogenic medium (DMEM/F12 medium (Sigma, USA) supplemented with 10% fetal bovine serum (FBS, Gibco, USA), 10 mmol/L sodium *β*-glycerophosphate, 0.05 mmol/L vitamin C, and 100 mmol/L dexamethasone (Sigma, USA)) for 2 weeks* in vitro*.

### 2.3. Procedure of Implantation

**Figure 1:**
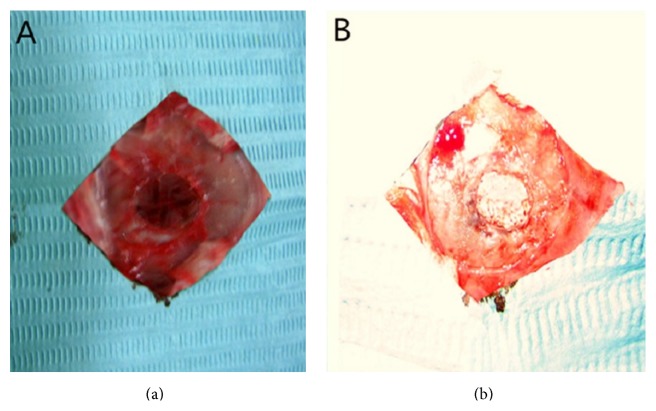
The images in calvarial defects of nude mice. (a) The calvarial defects of 6 mm in diameter. (b) The scaffolds were implanted into the calvarial defects.

Totally 108 nude mice (NIH strain, outbred, 8-week-old males) were preanesthetized with pentobarbital sodium (30 mg/kg) via intraperitoneal injection. The subcutaneous tissue, musculature, and periosteum were dissected to expose the calvarium. Full-thickness defects of 6 mm in diameter were created in the central areas of cranial bones using a saline-cooled trephine drill (Appledental, Hong Kong, China). After being maintained in an osteogenic medium for 2 weeks, the scaffolds seeded with hUCMSCs were implanted into the calvarial defects. Six groups were randomly divided according to the defects implanted with different contents: (1) PLGA scaffolds + hUCMSCs; (2) nHA/PLGA scaffolds + hUCMSCs; (3) CS/PLGA scaffolds + hUCMSCs; (4) nHA/CS/PLGA scaffolds + hUCMSCs; (5) nHA/CS/PLGA scaffolds without seeding; (6) the control group (no scaffolds) (*n* = 18, Figure 1). All animal experiments were approved by the Animal Care and Use Committee, School of Medicine, Xi'an Jiaotong University. All those surgeries were performed under anesthesia, with minimized damages to the mice.

### 2.4. Sequential Fluorescent Labeling and H&E Staining

In order to show the mineralization areas of new bones, sequential fluorescent labeling was performed by giving intracutaneous injections with xylenol orange (90 mg/kg, Xi'an Chemical Reagent Factory, China), fluorescein sodium (3 mg/kg, Xi'an Chemical Reagent Factory, China), and tetracycline (50 mg/kg, Xi'an Chemical Reagent Factory, China) on the back of nude mice at 1, 2, and 3 weeks after operation. At 4 weeks after operation, 12 nude mice from each group were euthanized by using the overdose of sodium pentobarbital, and the calvarial specimens with 2 mm of contiguous bones were then removed.

The nondecalcified calvarial specimens from the 6 nude mice in each group (*n* = 6) were made into the sections of 10 *μ*m in thickness. The sections were examined under the fluorescence microscope (Leica, Germany). The areas of new bones between the inner of red fluorescent bands and the lateral of yellow fluorescent bands were located and measured by Scion Image software (version beta 4.0.3). Measurements of all sections were performed 3 times by one researcher in this study. Fluorescence-labeled areas were expressed as the percentages of the total defect areas.

After decalcified in 14% ethylenediaminetetraacetic acid (EDTA) for 8 weeks, the 6 calvarial specimens from each group (*n* = 6) were embedded in paraffin and were made into H&E-stained sections. The sections were examined under an optical microscope (Olympus, Tokyo, Japan). The osteoid tissues and bone islands were located and measured by Scion Image software, expressed as the percentages of the total defect areas. Measurements of all sections were performed 3 times by one researcher in this study.

### 2.5. Van Gieson Staining

At 8 weeks, the remaining nude mice were euthanized, and the samples of six nude mice in each group were obtained by the above-mentioned method. Briefly, the nondecalcified samples were embedded by methyl methacrylate and cut into the sections of 80 *μ*m in thickness by a bone-sectioning machine (Malto, Japan). The sections were then stained with Van Gieson and examined under the optical microscope. The new bones were measured by Scion Image software, expressed as the percentages of the total defect areas. Measurements of all sections were performed 3 times by one researcher in this study.

### 2.6. Statistical Analysis

All data were analyzed using statistical software SPSS17.0 and expressed as means ± standard deviation. The significance tests were performed by one-way analysis of variance (ANOVA), followed by Bonferroni* post hoc* test. *p* value < 0.05 (*∗*) and *p* value < 0.01 (*∗∗*) were considered statistically significant.

## 3. Results

### 3.1. Sequential Fluorescent Labeling

**Figure 2:**
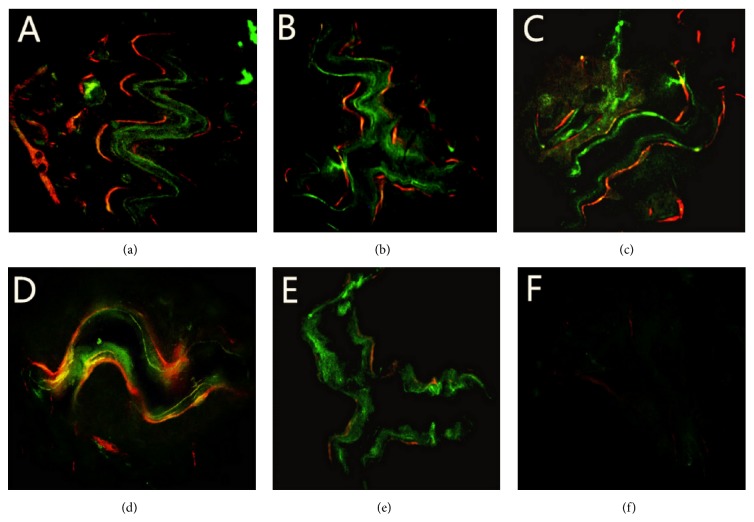
The images of sequential fluorescent labeling at 4 weeks. (a) nHA/CS/PLGA scaffolds + hUCMSCs; (b) nHA/PLGA scaffolds + hUCMSCs; (c) CS/PLGA scaffolds + hUCMSCs; (d) PLGA scaffolds + hUCMSCs; (e) nHA/CS/PLGA scaffolds without seeding; (f) the control group (no scaffolds). The fluorescent labeling was visible around and inside the defects and the fluorescence-labeled areas were more extensive compared with the other groups (*p* < 0.01) (a). Only weak fluorescence signals were found around the defects (f). Original magnification: 50x.

At 4 weeks, all nude mice acted and ate normally after operation. The operation areas showed mild swellings. The incisions were not infected and healed well. Rejections were not observed in all groups. The images of sequential fluorescent labeling exhibited fluorescent marks around the defects, except in the control group. Red, yellow, and green fluorescence bands were irregularly scattered as strips, rods, and bulks. No obvious boundaries were detected between those fluorescence bands. In the group of nHA/CS/PLGA scaffolds + hUCMSCs, the fluorescent labeling was visible at the edges and centers of the defects and the fluorescence-labeled areas showed more extensive signals compared with the other groups (*p* < 0.01) (Table 1). In contrast, only weak fluorescence signals were found around the defects in the control group (Figure 2).

### 3.2. Histological Examination

**Figure 3:**
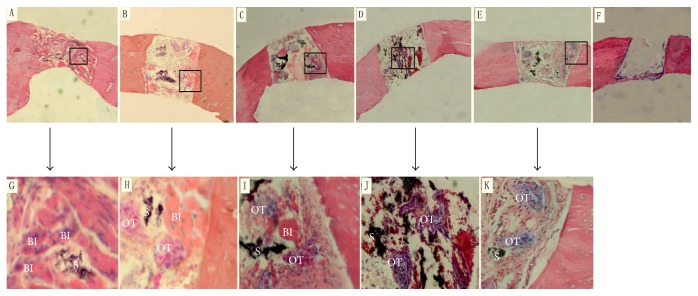
HE-stained sections of calvarial defects at 4 weeks. (A) nHA/CS/PLGA scaffolds + hUCMSCs; (B) nHA/PLGA scaffolds + hUCMSCs; (C) CS/PLGA scaffolds + hUCMSCs; (D) PLGA scaffolds + hUCMSCs; (E) nHA/CS/PLGA scaffolds without seeding; (F) the control group (no scaffolds). (G), (H), (I), (J), and (K) were the enlarged photographs of the rectangular frames in (A), (B), (C), (D), and (E), respectively. The bone islands were scattered around and inside the scaffolds (A). The bone islands could be occasionally observed around the defects. The osteoid tissues could be found around and inside the defects (B, C, and D). However, in the group of nHA/CS/PLGA scaffolds without seeding, osteoid tissues were only found around the defects, and bone islands were not detected (E). No osteoid tissues or bone islands were observed in the control group (F). BI: bone islands, OT: osteoid tissues, and S: scaffolds. Original magnification (A–F): 50x. Original magnification (G–K): 300x.

H&E-stained sections of calvarial defects at 4 weeks are shown in Figure 3. The scaffolds were partially degraded, and the residue of scaffolds and the loose fibrous connective tissue were found inside the scaffolds. In the group of nHA/CS/PLGA scaffolds + hUCMSCs, bone islands were scattered around and inside the defects. In the groups of nHA/PLGA scaffolds + hUCMSCs, CS/PLGA scaffolds + hUCMSCs, and PLGA scaffolds + hUCMSCs, bone islands could be occasionally observed around the defects. The osteoid tissues could be found around and inside the defects. However, in the group of nHA/CS/PLGA scaffolds without seeding, the osteoid tissues were only found around the defects, and the bone islands were not detected. No osteoid tissues or bone islands were observed in the control group (Figure 3). No infiltrated inflammatory cells were detected in all groups. It was shown in Table 2 that the percentages of the osteoid tissues and bone islands in the group of nHA/CS/PLGA + hUCMSCs were significantly higher than those of other groups (*p* < 0.01).

**Figure 4:**
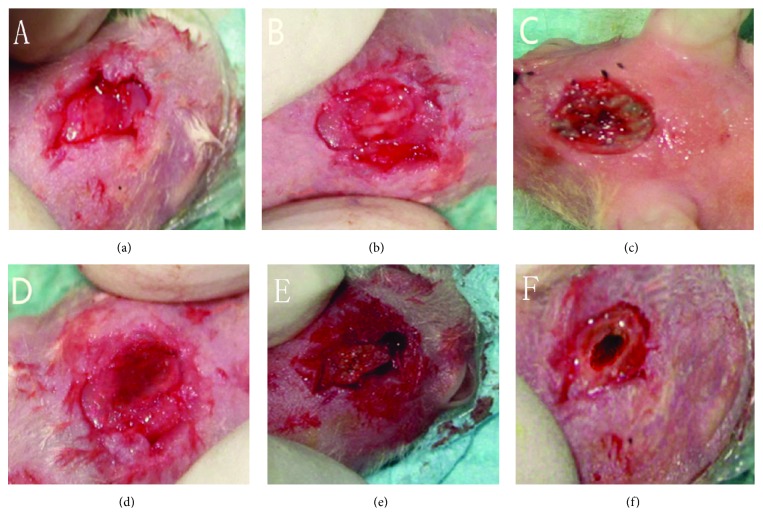
Macroscopic observation of the defects at 8 weeks after operation. (a) nHA/CS/PLGA scaffolds + hUCMSCs; (b) nHA/PLGA scaffolds + hUCMSCs; (c) CS/PLGA scaffolds + hUCMSCs; (d) PLGA scaffolds + hUCMSCs; (e) nHA/CS/PLGA scaffolds without seeding; (f) the control group (no scaffolds). The defects were filled with new bones (a). No obvious boundaries were found between the new bones and host bones (a–e). The defects were partially healed in the control group (f).

**Figure 5:**
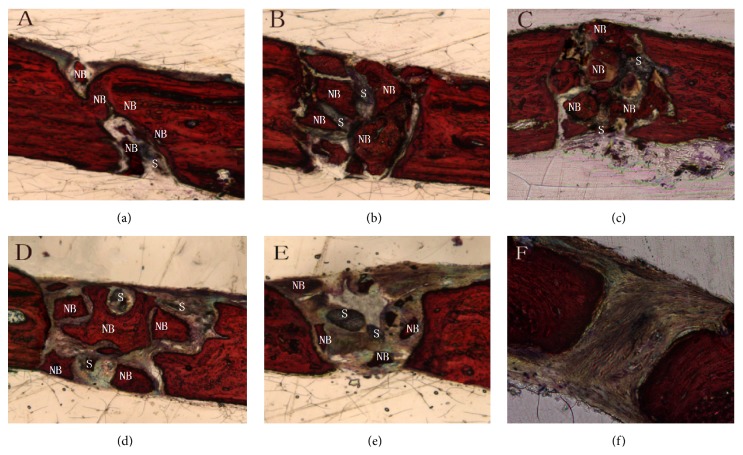
Van Gieson staining in the calvarial defects at 8 weeks. (a) nHA/CS/PLGA scaffolds + hUCMSCs; (b) nHA/PLGA scaffolds + hUCMSCs; (c) CS/PLGA scaffolds + hUCMSCs; (d) PLGA scaffolds + hUCMSCs; (e) nHA/CS/PLGA scaffolds without seeding; (f) the control group (no scaffolds). Most of bone defects were replaced by new bones and scaffolds were found degraded mostly. There was no difference between new bones and host bones. There were lots of mature and cord-like lamellar bones bridged with host bones (a). The remaining scaffolds could still be observed. New block-style and island-style bones formed inside the defects (b, c, d, and e). New bones were visible around the defects but invisible inside the defects (e). There were no new bones found in the control group (f). NB: new bone; S: scaffold. Original magnification: 300x.

At 8 weeks, in the group of nHA/CS/PLGA scaffolds + hUCMSCs, the defects were filled with new bones. No obvious boundaries were found between the new bones and host bones. In the other groups except for the control group, the defects were partially healed. Similarly, no obvious boundaries were found between the new bones and host bones (Figure 4). Van Gieson staining showed that most of bone defects were replaced by the new bones and scaffolds were largely degraded in the group of nHA/CS/PLGA + hUCMSCs. There was no difference between the new bones and host bones. There were lots of mature and cord-like lamellar bones bridged with the host bones. In the groups of nHA/PLGA scaffolds + hUCMSCs, CS/PLGA scaffolds + hUCMSCs, PLGA scaffolds + hUCMSCs, and nHA/CS/PLGA scaffolds without seeding, the remaining scaffolds could still be observed. New block-style and island-style bones were formed in the defect areas and the woven-shaped collagen fibers were coarse and disorderly arranged in these new bones. However, in the group of nHA/CS/PLGA scaffolds without seeding, new bones were visible around the defects, but invisible inside the defects (Figure 5).

New bones were the most in the group of nHA/CS/PLGA + hUCMSCs (*p* < 0.01) (Table 3). In the control group, the defects were not repaired and filled by connective tissues.

## 4. Discussion

Nude mice lack the thymus, resulting in the obstacle of generating T cells. Therefore, they are widely used in the experiments on immunology, oncology, toxicology, and other subjects of life science. Moreover, they have poor blood supply and bone regeneration. Immunosuppression of nude mice also causes their lack of key signals in the process of bone repairing, leading to retarded bone regeneration [14, 15]. So the calvarial defects of nude mice are good models for analyzing the repairing effect of scaffold materials on bone defects.

Sequential fluorescent labeling has been widely used in the researches on bone metabolism. The beginning of calcification and the formation of new bones are confirmed qualitatively by the method [16, 17]. In our study, the nude mice were injected sequentially with xylenol orange, fluorescein sodium, and tetracycline. Under the fluorescence microscope, fluorescent bands of xylenol orange were shown as bright red, fluorescein sodium as bright green, and tetracycline as bright yellow. By this simple method, the process of bone metabolism can be reflected.

Because the nondecalcified samples at 8 weeks were too hard to be made into fluorescent-labeled sections due to high degree of calcification, therefore the samples only at 4 weeks were made into the fluorescence-labeled sections. In the study, our data showed obvious difference by fluorescent markers between the group of nHA/CS/PLGA scaffolds + hUCMSCs and the other groups, suggesting that the formation of new bones in the group of nHA/CS/PLGA scaffolds + hUCMSCs was faster, compared with the other groups.

The bone samples for HE staining must be demineralized. The nucleus of cells at 8 weeks was stained as blue rather than red, because the samples at 8 weeks needed to be decalcified for a long time. So the sections of samples at 8 weeks were stained by Van Gieson instead of H&E.

It was reported that the scaffolds without seeding repaired bone defects mainly through bone conduction [18–20]. Firstly, the new vessels and bones grew from the edge to the inside of defects. In other words, bone repairing occurred from the joint of the host bones and scaffolds to the interior of scaffolds. However, the scaffolds seeded with cells repaired bone defects in two ways, that is, bone conduction and bone induction [21, 22]. New bones were formed around and inside the scaffolds. The main reason might be that the seeded cells in the scaffolds are proliferated and differentiated into osteoblasts, and new bones were formed finally. At the same time, the mesenchymal cells around the defects in the host bones are induced and differentiated into osteoblasts. It also facilitates forming new bones. In this experiment, it was shown that, in the group of nHA/CS/PLGA scaffolds without seeding, the repairing process occurred around the defects. At 8 weeks, the sections stained by Van Gieson showed the lack of new bones in the center of calvarial defects. In contrast, in the groups of scaffolds seeded with hUCMSCs, new bones were found around and inside the defects. Our findings suggested that osteogenesis of osteoblasts was promoted only by bone conduction around the defects in the group of nHA/CS/PLGA scaffolds without seeding. However, the conduction and induction of bones simultaneously occurred in the groups of nHA/CS/PLGA, nHA/PLGA, CS/PLGA, and PLGA scaffolds seeded with hUCMSCs. In addition, most of new bones were found in the group of nHA/CS/PLGA scaffolds + hUCMSCs. It was implied that nHA/CS/PLGA scaffolds contributed to osteogenic induction of hUCMSCs.

Degradability of scaffold materials is crucial for materials in bone tissue engineering. Growth factors secreted by seeded cells induce the aggregation of the osteoclasts via signal conduction. In addition, these growth factors lead to the degradation of materials [10]. In this study, it was shown in H&E staining at 4 weeks (Figure 4) and Van Gieson staining at 8 weeks (Figure 5) that nHA/CS/PLGA scaffolds were mostly degraded or broken down into smaller particles.

As a kind of ideal inorganic bioactive material, the particle sizes of nHA are small and its surface areas are large. Unique surface properties of nHA, such as chemical properties, surface topography, and surface energy, mediate the bioactivity of specific proteins, such as fibronectin, vitronectin, and laminin. Consequently, cell behaviors are regulated, and the tissue regenerations are further promoted [4, 23–25]. Therefore, nHA particles on the scaffolds might lead to higher mineralization and more new bones. CS is one of the most promising natural materials due to its remarkable physicochemical and biological properties. CS is widely available, and its hydrophilic surfaces can promote the adhesion, proliferation, and differentiation of cells [26–29]. It was reported that CS on the surface of PLGA scaffolds promoted the osteogenic differentiation of cells [30]. In addition, PLGA can be artificially synthesized with a well-controlled degradation rate, and it has been utilized in bone reconstruction due to its resorption and biocompatibility [31, 32]. Therefore, the three materials were usually chosen as raw materials of scaffolds. However, the compound of the three materials was not reported. In the previous study, we found that nHA/CS/PLGA scaffolds have better adhesion and proliferation for hUCMSCs, compared with nHA/PLGA, CS/PLGA, and PLGA scaffolds. The expression level of alkaline phosphatase and osteocalcin was higher* in vitro*. The formation of osteoid tissues was also more in subcutaneous experiment [7]. Because of poor mechanical properties and uncontrollable degradation properties, CS/nHA scaffolds were not included in our previous study. In this study, we found that the calvarial defects of nude mice were repaired more effectively by the nHA/CS/PLGA scaffolds seeded with hUCMSCs, compared with nHA/PLGA, CS/PLGA, and PLGA scaffolds seeded with hUCMSCs. There might be three reasons for the above process. (1) Bone repairing capability of scaffolds are magnified when inorganic materials, natural biomaterials, and synthetic polymer are combined, which was already reported in related researches [33–37]. (2) It was reported that the adhesion to the cells is improved after the surface areas of scaffolds are increased [38]. The particles of nHA and CS can increase the surface roughness of PLGA scaffolds, leading to better cell-adhesive capacity. (3) It was reported that better performance was achieved when the nHA/chitosan compounds were developed [39, 40]. Therefore, nHA and CS in nHA/CS/PLGA scaffolds probably had synthetic effects on repairing bone defects. However, the specific mechanism of repairing bone defects for nHA/CS/PLGA scaffolds seeded with hUCMSCs needs to be further investigated.

It was reported that it was difficult to repair critical calvarial defects in a short time [40]. Therefore, it was crucial to accelerate the repairing of calvarial defects. In the group of nHA/CS/PLGA scaffolds + hUCMSCs, we found the areas of osteoid tissues and bone tissues were the largest at 4 and 8 weeks, which was statistically significant. It was implied that nHA/CS/PLGA scaffolds seeded with hUCMSCs could accelerate the bone regeneration at early stage.

## 5. Conclusion

Our findings revealed that nHA and CS enhanced the bone regeneration of nHA/CS/PLGA scaffolds seeded with hUCMSCs in the calvarial defects of the nude mice at early stage. nHA/CS/PLGA scaffolds seeded with hUCMSCs could have wide applications in bone tissue engineering.

## Supplementary Material

The original data of experimental results before statistical analysis are showned in Table 1–3.

## Figures and Tables

**Table 1:** The percentages of fluorescence-labeled areas at 4 weeks after operation (*n* = 6).

Groups	nHA/CS/PLGA scaffolds + hUCMSCs	nHA/PLGA scaffolds + hUCMSCs	CS/PLGA scaffolds + hUCMSCs	PLGA scaffolds + hUCMSCs	nHA/CS/PLGA scaffolds without seeding	The control group
The percentages of fluorescence-labeled areas (%)	46.33 ± 9.07^*∗∗*^	31.66 ± 18.72	25.33 ± 8.73	21.33 ± 7.02	11.00 ± 4.58	1.67 ± 3.78

The percentages (%) of fluorescence-labeled areas in nHA/CS/PLGA scaffolds + hUCMSCs group were higher than those of other groups, ^*∗∗*^ *p* < 0.01.

**Table 2:** The percentages of the osteoid tissues and bone islands at 4 weeks (*n* = 6).

Groups	nHA/CS/PLGA scaffolds + hUCMSCs	nHA/PLGA scaffolds + hUCMSCs	CS/PLGA scaffolds + hUCMSCs	PLGA scaffolds + hUCMSCs	nHA/CS/PLGA scaffolds without seeding	The control group
The percentages of the osteoid tissues and bone islands (%)	51.50 ± 4.69^*∗∗*^	32.63 ± 3.79	28.73 ± 3.18	27.09 ± 3.23	21.32 ± 2.88	0

The percentages (%) of the osteoid tissues and bone islands in nHA/CS/PLGA scaffolds + hUCMSCs group were higher than those of other groups, ^*∗∗*^ *p* < 0.01.

**Table 3:** The percentages of new bones at 8 weeks (*n* = 6).

Groups	nHA/CS/PLGA scaffolds + hUCMSCs	nHA/PLGA scaffolds + hUCMSCs	CS/PLGA scaffolds + hUCMSCs	PLGA scaffolds + hUCMSCs	nHA/CS/PLGA scaffolds without seeding	The control group
The percentages of new bones (%)	74.30 ± 6.52^*∗∗*^	61.66 ± 2.73	55.29 ± 8.76	49.46 ± 6.21	32.23 ± 4.67	0

The area percentages (%) of new bones in nHA/CS/PLGA scaffolds + hUCMSCs group were higher than those of the other groups, ^*∗∗*^ *p* < 0.01.
