# Supplementary material for: Bone Regeneration by Nanohydroxyapatite/Chitosan/Poly(lactide-co-glycolide) Scaffolds Seeded with Human Umbilical Cord Mesenchymal Stem Cells in the Calvarial Defects of the Nude Mice
Source: Biomed Res Int. 2015 Oct 13;2015:261938. doi: 10.1155/2015/261938 (PMC4621339; doi:10.1155/2015/261938)
Supplement: Supplementary file 1 — The original data of experimental results before statistical analysis are showned in Table 1–3. [file 261938.f1.doc]

**original data**

Table 1 The percentages(%) of fluorescence-labeled areas at 4 weeks after operation.（n=6）

|  | nHA/CS/PLGA  + hUCMSCs | nHA/PLGA+ hUCMSCs | CS/PLGA+ hUCMSCs | PLGA+ hUCMSCs | nHA/CS/PLGA witout seeding | the control group |
| --- | --- | --- | --- | --- | --- | --- |

| 1 | 56.02 | 33.23 | 23.32 | 20.06 | 9.14 | 0.00 |
| --- | --- | --- | --- | --- | --- | --- |
| 2 | 55.24 | 26.34 | 24.41 | 27.47 | 14.93 | 1.96 |
| 3 | 57.32 | 32.49 | 23.01 | 28.82 | 11.02 | 0.40 |
| 4 | 38.14 | 18.04 | 36.13 | 14.31 | 12.29 | 1.13 |
| 5 | 37.12 | 17.04 | 26.21 | 14.67 | 11.15 | 2.97 |
| 6 | 39.02 | 18.92 | 25.78 | 15.01 | 13.93 | 1.84 |
| 7 | 45.34 | 24.21 | 19.06 | 22.12 | 7.31 | 0.00 |
| 8 | 44.19 | 24.89 | 29.34 | 22.84 | 14.96 | 0.92 |
| 9 | 46.37 | 23.57 | 38.25 | 21.69 | 11.36 | 0.52 |
| 10 | 47.13 | 33.29 | 22.53 | 31.32 | 8.32 | 0.00 |
| 11 | 50.24 | 26.72 | 28.94 | 29.36 | 17.74 | 0.42 |
| 12 | 46.21 | 28.81 | 26.36 | 24.19 | 12.39 | 0.21 |
| 13 | 44.97 | 25.19 | 28.38 | 19.28 | 18.20 | 0.90 |
| 14 | 47.32 | 19.93 | 19.94 | 27.81 | 13.27 | 0.13 |
| 15 | 52.18 | 21.98 | 31.47 | 31.28 | 11.28 | 2.28 |
| 16 | 50.26 | 23.19 | 25.19 | 28.16 | 15.29 | 0.00 |
| 17 | 49.15 | 25.63 | 28.38 | 23.17 | 8.33 | 0.14 |
| 18 | 41.39 | 27.42 | 24.29 | 25.94 | 9.74 | 1.29 |

Table 2 The percentages（%）of the osteoid tissues and bone islands at 4 weeks after operation.（n=6）

|  | nHA/CS/PLGA  + hUCMSCs | nHA/PLGA+ hUCMSCs | CS/PLGA+ hUCMSCs | PLGA+ hUCMSCs | nHA/CS/PLGA witout seeding | the control group |
| --- | --- | --- | --- | --- | --- | --- |
| 1 | 48.03 | 23.56 | 21.54 | 19.36 | 14.32 | 0 |
| 2 | 47.45 | 22.45 | 27.41 | 18.92 | 23.24 | 0 |
| 3 | 49.15 | 24.69 | 22.36 | 20.14 | 15.62 | 0 |
| 4 | 62.93 | 35.21 | 30.13 | 29.37 | 23.45 | 0 |
| 5 | 60.34 | 34.79 | 29.44 | 28.14 | 22.62 | 0 |
| 6 | 63.98 | 36.27 | 31.28 | 30.36 | 34.26 | 0 |
| 7 | 43.54 | 39.13 | 34.52 | 32.42 | 26.21 | 0 |
| 8 | 42.46 | 28.45 | 33.25 | 29.87 | 15.37 | 0 |
| 9 | 44.48 | 40.37 | 25.17 | 33.42 | 27.37 | 0 |
| 10 | 45.56 | 29.12 | 28.73 | 22.15 | 22.36 | 0 |
| 11 | 46.33 | 32.54 | 35.21 | 26.29 | 23.46 | 0 |
| 12 | 50.12 | 35.29 | 26.37 | 21.35 | 18.18 | 0 |
| 13 | 58.26 | 39.38 | 29.14 | 29.17 | 29.12 | 0 |
| 14 | 52.19 | 30.19 | 29.47 | 26.36 | 23.97 | 0 |
| 15 | 46.26 | 29.75 | 22.52 | 27.38 | 18.26 | 0 |
| 16 | 54.71 | 33.29 | 28.39 | 25.42 | 21.21 | 0 |
| 17 | 58.14 | 35.29 | 32.57 | 23.31 | 22.45 | 0 |
| 18 | 50.21 | 30.14 | 22.38 | 24.89 | 25.38 | 0 |

Table 3 The area percentages（%）of new bones in each group at 8 weeks.（n=6）

|  | nHA/CS/PLGA  + hUCMSCs | nHA/PLGA+ hUCMSCs | CS/PLGA+ hUCMSCs | PLGA+ hUCMSCs | nHA/CS/PLGA witout seeding | the control group |
| --- | --- | --- | --- | --- | --- | --- |
| 1 | 68.34 | 63.98 | 65.34 | 51.63 | 27.93 | 0 |
| 2 | 67.21 | 62.16 | 64.24 | 49.27 | 29.03 | 0 |
| 3 | 69.27 | 64.27 | 66.25 | 53.16 | 26.36 | 0 |
| 4 | 73.31 | 58.65 | 51.28 | 42.46 | 37.21 | 0 |
| 5 | 72.18 | 57.37 | 50.36 | 43.58 | 38.79 | 0 |
| 6 | 74.26 | 56.34 | 52.47 | 40.97 | 36.25 | 0 |
| 7 | 81.26 | 62.35 | 49.25 | 54.29 | 31.55 | 0 |
| 8 | 79.25 | 65.34 | 50.91 | 53.27 | 32.74 | 0 |
| 9 | 82.47 | 60.24 | 48.14 | 55.38 | 29.86 | 0 |
| 10 | 67.23 | 55.04 | 46,24 | 46.23 | 28.92 | 0 |
| 11 | 73.13 | 56.25 | 47.26 | 48.26 | 29.33 | 0 |
| 12 | 83.25 | 59.18 | 50.15 | 51.35 | 35.62 | 0 |
| 13 | 77.26 | 61.23 | 49.24 | 49.23 | 33.94 | 0 |
| 14 | 73.97 | 63,16 | 48.95 | 41.32 | 31.25 | 0 |
| 15 | 79.16 | 59,43 | 58.26 | 46.26 | 30.15 | 0 |
| 16 | 70.28 | 58.18 | 53.47 | 44.26 | 27.93 | 0 |
| 17 | 72.93 | 62.93 | 55.28 | 48.92 | 29.91 | 0 |
| 18 | 74.64 | 58.38 | 58.25 | 51.26 | 27.03 | 0 |
